# Dual Fluorescence–Lipid Endpoints Resolve Species- and Metal-Specific Toxicity Patterns in Marine Diatoms

**DOI:** 10.3390/toxics14030267

**Published:** 2026-03-18

**Authors:** Hojun Lee, Taejun Han, Jihae Park

**Affiliations:** 1 Marine@UGent Korea, Ghent University Global Campus, 119-5, Songdomunhwa-ro, Incheon 21985, Republic of Korea; hojun.lee@ugent.be (H.L.); taejun.han@ghent.ac.kr (T.H.); 2 Bio Environmental Science and Technology (BEST) Lab, Ghent University Global Campus, 119-5, Songdomunhwa-ro, Incheon 21985, Republic of Korea; 3 Department of Animal Sciences and Aquatic Ecology, Ghent University, Wetenschapspark 1, Bluebridge, 8400 Oostende, Belgium; 4 Center for Green Chemistry and Environmental Biotechnology, Ghent University Global Campus, 119-5 Songdomunhwa-ro, Yeonsu-gu, Incheon 21985, Republic of Korea

**Keywords:** *Cylindrotheca closterium*, nile red lipid assay, sublethal biomarkers, *Thalassiosira weissflogii*, trace metal toxicity, fluorescence-based bioassay

## Abstract

Trace metals are persistent stressors in coastal ecosystems, yet most marine algal toxicity assessments still rely on freshwater model species and growth-based endpoints that provide limited mechanistic resolution. Here, we quantified the sensitivity of two ecologically contrasting marine diatoms—the benthic *Cylindrotheca closterium* and the planktonic *Thalassiosira weissflogii*—to ten environmentally relevant metals using a dual-endpoint approach that integrates chlorophyll fluorescence (photosystem function) and Nile Red-based lipid-body fluorescence (metabolic reallocation). Fluorescence-based EC_10_ values revealed distinct species- and metal-specific patterns, with *C. closterium* consistently responding at lower concentrations and Hg producing the strongest inhibition in both species (EC_10_ ≈ 0.04–0.06 mg L^−1^). Lipid-body accumulation detected earlier metabolic disturbance for several metals, particularly Hg, As, Cr(VI), and Cd, and frequently occurred at concentrations where fluorescence remained minimally affected. These sequential thresholds indicate that pigment impairment and metabolic reallocation represent mechanistically distinct stages of the cellular stress response that differ among metals and between diatom guilds. Comparison with published toxicity data shows that the dual-endpoint sensitivities observed here fall within, or slightly above, the upper range of reported microalgal responses, underscoring the pronounced susceptibility of benthic diatoms to redox-active and thiol-reactive metals. The strong agreement between fluorescence-based EC values and traditional growth-derived benchmarks for key metals further supports fluorescence as an operationally efficient endpoint suitable for integration into emerging ISO marine algal bioassays. Overall, this study demonstrates that pairing a rapid functional marker with a mechanistically informative metabolic biomarker enables metal-specific toxicity fingerprinting and provides an ecologically grounded basis for incorporating benthic diatoms into coastal metal risk assessment frameworks.

## 1. Introduction

Marine diatoms are key primary producers in coastal ecosystems and among the first organisms to experience and integrate chemical changes in the water column and sediment–water interface [1]. Because they occur at the functional junction of pelagic and benthic carbon cycling, diatoms can act not only as “test organisms” but also as bioindicators that reflect both contaminant exposure and associated changes in photosynthetic activity and metabolic reorganization. Their high surface-area-to-volume ratio, rapid growth, and direct exposure to dissolved and particulate contaminants make them particularly responsive to trace metal pollution. Benthic diatoms colonizing sediment surfaces encounter metals in resuspended particles and porewater, whereas planktonic diatoms in the water column are primarily exposed to dissolved metal species. This ecological contrast provides a framework for understanding how habitat and life history influence sensitivity to anthropogenic contamination [2,3].

Despite the ecological prominence of diatoms, most standard algal toxicity tests used in regulation focus on freshwater green algae with cell density-based growth inhibition as the primary endpoint. The Organization for Economic Cooperation and Development OECD Test Guideline 201 [4] and related International Organization for Standardization (ISO) guidelines [5] require repeated cell counts or optical density measurements used as a proxy for cell density over several days, which are relatively labor-intensive, time-consuming, and not easily automated. These constraints limit ecotoxicological testing throughput and its integration into routine monitoring schemes [6]. Additionally, the dependence on a single growth endpoint captures only the final outcome of stress, providing limited mechanistic insight into the effects of contaminants on cellular physiology before biomass decline. This is a critical limitation for coastal risk assessment, where exposures are often low-level and chronic but may also occur as short contamination pulses. Under such conditions, early functional impairment and metabolic stress can precede detectable growth inhibition. For marine systems, where ionic strength, complexation, and speciation differ markedly from those in freshwater environments, ecologically relevant and operationally efficient test systems are urgently required [7,8].

In response to these limitations, fluorescence-based approaches have emerged as promising alternatives to traditional growth measurements [9,10]. Bulk fluorescence intensity, derived from algal cell pigments, can serve as a rapid proxy for biomass, enabling quantitative assessment of algal inhibition within seconds using simple fluorometric devices [11]. As this signal can be obtained non-destructively at high temporal resolution, fluorescence enables automation and high-throughput screening, in contrast to manual cell counting. Recent methodological developments, including proposals from German working groups to the ISO marine algae community, have suggested replacing conventional growth endpoints with fluorescence-based biomass measurements to modernize and simplify marine algal toxicity tests [5,12]. However, for fluorescence to serve as a regulatory-grade endpoint, it must be validated across ecologically relevant taxa (including marine diatoms), across diverse metal modes of action, and against endpoints that capture sublethal stress that may not manifest as biomass loss within standard test durations [13,14]. If validated across relevant taxa and stressors, this substitution could reduce analytical effort while preserving, or even improving, sensitivity to toxic effects.

Simultaneously, it is increasingly recognized that population-level endpoints or their proxies cannot fully characterize the impact of contaminants on primary producers. Sublethal biomarkers that report on cellular stress and metabolic reorganization can reveal “hidden” toxicity preceding or occurring independently of changes in biomass. Neutral lipid accumulation, visualized and quantified using fluorescent dyes such as Nile Red, is one such biomarker [15,16]. Under chemical stress, microalgae redirect carbon from growth toward storage lipids, both as a buffer against energy imbalance and to sequester damaged components. Metal exposure induces oxidative stress and perturbs central metabolism, promoting lipid body formation. Measuring lipid accumulation alongside fluorescence-based biomass proxies provides a compact yet mechanistically informative endpoint pair: capturing overall population performance and sublethal metabolic stress, respectively. Critically, this dual-endpoint logic creates a pathway from “screening” toward “mechanism-informed prioritization,” enabling early identification of hazardous exposure regimes even when growth inhibition is subtle.

Metals are among the most persistent and tightly regulated contaminants in coastal waters, and are targeted by regional and international frameworks, such as the EU Water Framework Directive [17], US EPA priority pollutant lists [18], and various marine protection conventions [19]. Metals do not degrade but instead cycle between dissolved and particulate phases, accumulating in sediments and re-entering the water column through resuspension and bioturbation [20,21]. Consequently, coastal phytobenthos and phytoplankton experience chronic, low-level exposure superimposed on episodic pulses following discharge or disturbance events. Metals such as mercury, copper, arsenic, and cadmium interfere directly with photosynthetic pigments, membrane integrity, redox balance, and enzyme function at relatively low concentrations, making them potent stressors for microalgae and the base of marine food webs [22,23]. Because many metal stress pathways converge on photosynthetic function and carbon allocation, metals provide stringent test cases for evaluating whether fluorescence inhibition (functional biomass proxy) and lipid-body accumulation (metabolic stress proxy) can generate distinct, interpretable “toxicity fingerprints.” As these metals act through well-defined physiological pathways directly impacting pigments and cellular metabolism, they provide stringent and informative test cases for assessing fluorescence- and lipid-based endpoints.

**Figure 1:**
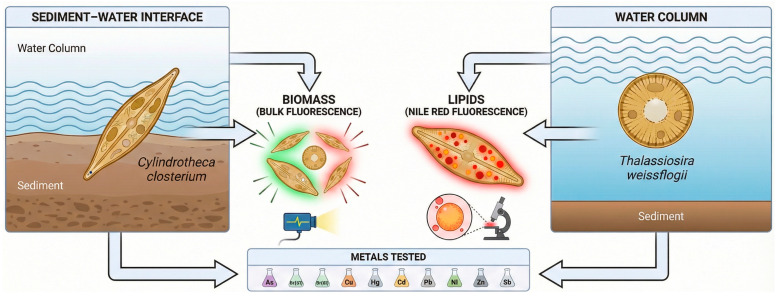
Conceptual overview of the study framework. Two marine diatoms were used: the benthic species *Cylindrotheca closterium* and the planktonic species *Thalassiosira weissflogii*. Two rapid endpoints were evaluated: bulk fluorescence as a proxy for biomass and Nile Red fluorescence as an indicator of lipid-body accumulation. The responses of the two species were assessed across ten metals (As, Cr(VI), Cr(III), Cu, Hg, Cd, Pb, Ni, Zn, Sb).

For each metal–species combination, we quantified concentration–response relationships using two endpoints: a bulk fluorescence signal as a rapid proxy for biomass inhibition, and Nile Red-derived lipid body fluorescence as a measure of sublethal metabolic stress. By explicitly pairing two diatom guilds with two mechanistically complementary endpoints, we aim to move beyond single-endpoint growth inhibition toward a streamlined, automation-ready framework that is both ecologically grounded and standardization-compatible (Figure 1).

By deriving EC_10_ and EC_50_ values for both endpoints across two contrasting diatom guilds, we addressed three main questions. First, how do benthic and planktonic diatoms differ in their quantitative sensitivity to individual metals when assessed using rapid fluorescence-based methods? Second, to what extent does lipid body accumulation reveal stress at concentrations where fluorescence-based biomass inhibition is weak or absent, and does this pattern vary among metals? Third, how can these dual-endpoint, dual-guild data inform the development of streamlined, ISO-compatible marine algal bioassays that reflect the realities of coastal metal contamination? Addressing these questions provides novel insights into the physiology of diatom responses to trace metals and offers an actionable blueprint for integrating rapid fluorescence and metabolic biomarkers into next-generation regulatory and monitoring-oriented toxicity screening in marine environments.

## 2. Materials and Methods

### 2.1. Test Organisms and Culture Conditions

Two marine diatom species representing contrasting ecological niches and exposure pathways were used: the benthic pennate diatom *Cylindrotheca closterium* (strain LIMS-PS-0616) and the planktonic centric diatom *Thalassiosira weissflogii*. The *C. closterium* strain was obtained from the Korea Institute of Ocean Science & Technology (KIOST) Library of Marine Samples, whereas *T. weissflogii* was purchased from the National Center for Marine Algae and Microbiota (NCMA), Bigelow Laboratory for Ocean Sciences, USA.

Both strains were maintained in 100 mL culture flasks (SPL Life Sciences, Pocheon, Republic of Korea) containing f/2 medium supplemented with silica, prepared using 0.47 µm filtered artificial seawater according to Guillard and Ryther [24]. Vitamins and carbonates were added prior to autoclave sterilization to ensure nutrient stability. The salinity was adjusted to 33 psu, and pH was maintained at 8.0 throughout cultivation.

Cultures were acclimatized for at least 20 generations under identical environmental conditions to minimize physiological drift and harmonize baseline growth and pigment status prior to exposure. Incubation was conducted at 18 °C under a photon flux density (PFD) of 30 ± 2 µmol photons m^−2^ s^−1^ with a 12:12 h light–dark cycle. Illumination was provided by square LED panels (340 mm × 500 mm × 10 mm; Daewon, Bucheon, Republic of Korea) equipped with 20 diodes per strip spaced 1 cm apart. Uniform light distribution was ensured using neutral-density shading layers, and photon flux density was verified with a quantum sensor (LI-1400, LI-COR, Lincoln, NE, USA). The experimental design used standardized laboratory light conditions to ensure reproducible toxicity comparisons rather than to reproduce natural light gradients occurring in aquatic ecosystems.

Cultures were gently swirled by hand once daily to prevent sedimentation while avoiding shear stress. Only cells in the mid-exponential growth phase—typically 4–5 days after inoculation—were used for toxicity testing to ensure comparable physiological states at the onset of metal exposure.

### 2.2. Metal Preparation and Exposure Solutions

Ten trace metals were selected: As, Cd, Cr(III), Cr(VI), Cu, Hg, Ni, Pb, Sb, and Zn. Certified 1000 mg L^−1^ (ppm) standard solutions for each metal were purchased from a commercial supplier. All standards were supplied in acidified aqueous matrices (trace metal grade) to ensure stability.

Working exposure solutions were prepared immediately prior to each experiment by appropriate dilution of the stock standards in sterile artificial seawater enriched with f/2 nutrients. Final acid concentrations resulting from stock dilution were negligible and were verified not to affect fluorescence- or lipid-based endpoints.

Solvent controls were not required, as no organic solvents were used. Concentration ranges were determined through preliminary range-finding tests to ensure that complete concentration–response relationships could be resolved within the 48 h exposure period without inducing confounding nutrient limitation or optical self-shading effects.

### 2.3. Exposure Design and Experimental Conditions

All toxicity assays were conducted using a 48 h static exposure design. Each test was performed in acid-washed borosilicate glass flasks filled with 50 mL of exposure medium. Initial cell densities were standardized at 5 × 10^4^ cells mL^−1^ for *C. closterium* and 2 × 10^4^ cells mL^−1^ for *T. weissflogii*. The lower initial density for *T. weissflogii* reflects its larger cell size and higher chlorophyll content per cell, as confirmed in preliminary calibration tests demonstrating species-specific differences in fluorescence yield per cell. Density selection ensured that both species remained within the linear fluorescence detection range throughout the 48 h exposure while preventing optical self-shading and nutrient limitation.

Flasks were randomly arranged within the incubation chamber to minimize potential bias caused by subtle gradients in light or temperature. Environmental conditions during exposure were identical to those used for culture maintenance. Cultures were gently swirled twice daily to maintain homogeneity of suspended cells. Temperature, pH, salinity, and dissolved oxygen were recorded at 48 h to confirm environmental stability and to ensure that physicochemical drift did not confound biological responses. Seawater and solvent controls were included in every experiment, and each concentration was tested in triplicate biological replicates.

### 2.4. Fluorescence-Based Biomass Quantification

Fluorescence was used as a rapid, non-destructive proxy for algal biomass. Measurements were performed directly in the 24-well microplates used for exposure to avoid sample transfer and potential settling artifacts. At the end of the 48 h exposure period, plates were gently shaken to ensure homogeneity prior to measurement, thereby maintaining consistent optical geometry across wells.

Fluorescence was measured using a microplate fluorometer (Gemini EM, Molecular Devices, San Jose, CA, USA) with excitation and emission wavelengths set at 485 and 680 nm, respectively, corresponding to chlorophyll-associated bulk fluorescence. Preliminary calibration experiments confirmed that fluorescence intensity was linearly correlated with cell density within the concentration range applied in toxicity testing.

Each biological replicate was measured in triplicate technical readings, and the averaged value was used for subsequent analysis. Percentage inhibition was calculated relative to the mean fluorescence of the corresponding control group.

### 2.5. Determination of Lipid Body Accumulation

Lipid body accumulation was quantified using Nile Red staining, a fluorescent dye selective for intracellular neutral lipid bodies. At the end of the 48 h exposure period, culture suspensions were gently homogenized in the original 24-well plates, and 297 µL aliquots from each well were transferred to black 96-well microplates.

A 3 µL aliquot of Nile Red stock solution (0.1 mg mL^−1^ in acetone; Sigma-Aldrich, St. Louis, MO, USA) was added to each well [16], resulting in a final volume of 300 µL and a final dye concentration of 1 µg mL^−1^. Samples were gently mixed and incubated in darkness at room temperature for 20 min to allow consistent intracellular staining while minimizing photo-oxidation.

Fluorescence was recorded using a microplate reader (Gemini EM, Molecular Devices, San Jose, CA, USA) with excitation and emission wavelengths set at 530 and 592 nm, respectively. Background fluorescence from dye-only controls was subtracted prior to analysis.

Because Nile Red fluorescence depends on cell abundance, lipid fluorescence values were normalized to the corresponding bulk chlorophyll fluorescence of each sample to express lipid accumulation on a per-biomass basis.

### 2.6. Data Analysis and Statistical Treatment

Concentration–response relationships for both fluorescence-based biomass inhibition and lipid-body accumulation were modeled using a three-parameter logistic regression implemented in SigmaPlot version 12.5 (Systat Software Inc., San Jose, CA, USA). EC_10_ and EC_50_ values were estimated from fitted curves together with their 95% confidence intervals (CIs). Goodness-of-fit was evaluated by examining residual distributions and coefficients of determination (R^2^).

All fluorescence and lipid data were expressed relative to the corresponding control means prior to modeling. Lipid fluorescence values were normalized to bulk chlorophyll fluorescence to account for biomass-dependent signal variation.

Differences among treatment groups were assessed using one-way analysis of variance (ANOVA). Prior to hypothesis testing, homogeneity of variance was verified to ensure the validity of parametric analyses. Post hoc pairwise comparisons were performed using the least significant difference (LSD) test at a significance level of α = 0.05. Sensitivity comparisons between species and endpoints were based exclusively on EC values obtained under identical experimental conditions.

## 3. Results

### 3.1. Fluorescence-Based Biomass Inhibition: Species- and Metal-Specific Sensitivity

**Figure 2:**
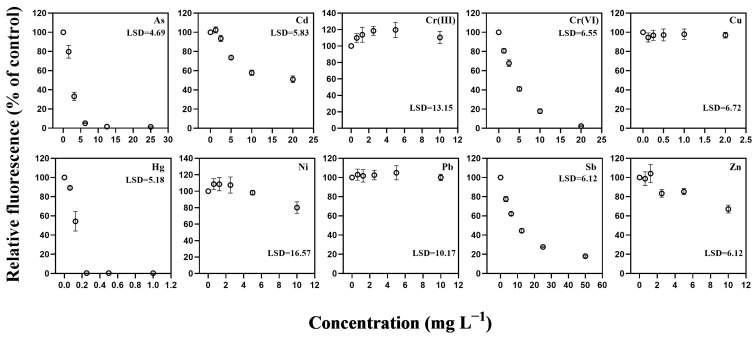
Concentration–response relationships for fluorescence-based biomass inhibition in *Cylindrotheca closterium* after 48 h exposure to ten trace metals. Relative fluorescence (% of control) is shown as mean ± SE (*n* = 4). Data points represent mean values, and error bars indicate standard error. Panels correspond to As, Cd, Cr(III), Cr(VI), Cu, Hg, Ni, Pb, Sb, and Zn. Lower fluorescence indicates stronger biomass inhibition. Concentrations are expressed in mg L^−1^.

Cd displayed a more gradual decline, with significant reductions emerging only at mid-range concentrations, consistent with its comparatively high EC_10_ and EC_50_ values (Figure 2 and Table 1). In contrast, Cr(III), Ni, Pb, and Cu produced only weak or delayed inhibition within the 48 h exposure period, with EC_10_ or EC_50_ values exceeding the upper limits of the tested concentration range. Sb and Zn induced clearer inhibition, with EC_10_ values of 1.394 and 1.419 mg L^−1^, respectively, although neither metal reached complete suppression of fluorescence within 48 h. These outcomes indicate substantial variation in potency among metals, with Hg, As, and Cr(VI) producing the strongest inhibition and several others exhibiting only partial or gradual effects.

The behavior of Cd and Cr(III) in *C. closterium* contrasted with the sharp declines observed for Hg, As, and Cr(VI). Cd produced a moderate and gradual reduction in fluorescence, with inhibition emerging at mid-range concentrations and EC_10_ and EC_50_ values of 2.8265 and 5.416 mg L^−1^, respectively (Table 1). Cr(III) produced no measurable inhibition within the tested range, with both EC_10_ and EC_50_ values exceeding the upper limits of the exposure concentrations. Several other metals, including Ni, Pb, Sb, and Zn, also failed to reach complete inhibition within 48 h, leading to unresolved EC_50_ values (>10 mg L^−1^). These patterns indicate that these metals exert comparatively weaker acute effects on pigment-associated biomass in *C. closterium* under the present exposure conditions.

**Figure 3:**
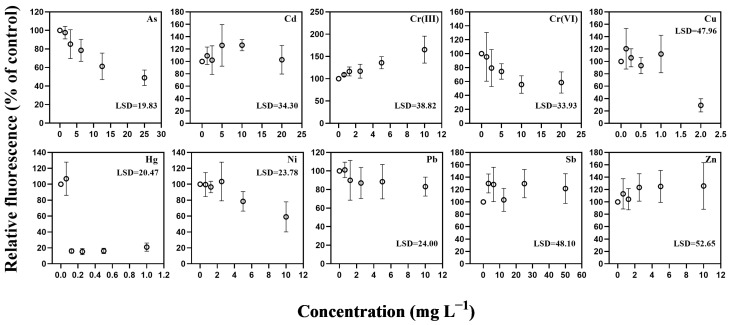
Concentration–response relationships for fluorescence-based biomass responses in *Thalassiosira weissflogii* after 48 h exposure to ten trace metals. Relative fluorescence (% of control) is presented as mean ± SE (*n* = 4). Data points represent mean values, and error bars indicate standard error. Panels correspond to As, Cd, Cr(III), Cr(VI), Cu, Hg, Ni, Pb, Sb, and Zn. Concentrations are expressed in mg L^−1^.

Applying the same fluorescence endpoint to the planktonic centric diatom *T. weissflogii* revealed a mixture of convergent and divergent patterns (Figure 3). Hg produced the strongest inhibition in this species as well, with fluorescence declining sharply at low concentrations and yielding an EC_10_ of 0.0760 mg L^−1^ and an EC_50_ of 0.1235 mg L^−1^ (Table 1). These thresholds closely matched those observed for *C. closterium*, indicating that Hg impairs pigment-associated biomass at similarly low concentrations regardless of ecological guild. For As, substantially higher concentrations were required to elicit comparable inhibition, with EC_10_ and EC_50_ values of 2.613 mg L^−1^ and 8.291 mg L^−1^, respectively. Cr(VI) produced measurable reductions in fluorescence at lower concentrations, but inhibition did not progress sufficiently at higher exposure levels to yield a resolvable EC_50_, resulting in a less progressive dose–response pattern than that observed in the benthic species.

Cu produced highly variable fluorescence responses in *T. weissflogii* (Figure 3), with values fluctuating around the control across most concentrations and significant inhibition detected only at the highest exposure level (2 mg L^−1^). Consistent with this pattern, no resolvable EC_10_ or EC_50_ values could be obtained within the tested range (Table 1). Several other metals, including Ni, Zn, and Pb, also failed to reach complete inhibition within 48 h, resulting in unresolved EC_50_ values. Cr(III) produced elevated or variable fluorescence rather than clear inhibition, likewise preventing the estimation of EC_10_ and EC_50_. Together, these responses indicate that these metals exerted incomplete or inconsistent acute effects on pigment-associated fluorescence in this species under the present exposure conditions.

Across both species, Hg produced consistently low EC_10_ and EC_50_ values, with thresholds clustering below 0.1–0.2 mg L^−1^, confirming its strong inhibitory effect on pigment-associated fluorescence regardless of ecological guild. Responses to other metals diverged more clearly between species. For As, *C. closterium* responded at substantially lower concentrations than *T. weissflogii*, whereas Cr(VI) induced measurable inhibition in both species but did not progress sufficiently in the planktonic species to yield a resolvable EC_50_ within the tested range.

In *C. closterium*, Cd, Ni, and Pb produced moderate or incomplete inhibition within 48 h, while Cr(III) caused no measurable inhibition. Sb and Zn also induced clear reductions in fluorescence, although neither metal reached complete suppression, resulting in unresolved EC_50_ values. In *T. weissflogii*, Cd, Cr(III), Cu, and Zn frequently produced variable or stimulatory responses, contributing to unresolved EC values. Cr(VI) and Sb elicited stronger and more progressive inhibition in the benthic species than in the planktonic species. Collectively, these patterns highlight substantial species-specific differences in sensitivity and response stability across metals.

### 3.2. Lipid-Body Accumulation: An Early Indicator of Metabolic Stress

Lipid-body accumulation, quantified via Nile Red fluorescence, provides insight into metabolic adjustments that complement and often precede fluorescence-based biomass inhibition. Interpreted alongside fluorescence, lipid-body formation functions as a compact “stress-allocation” readout reflecting carbon reprogramming under chemical challenge. In *C. closterium*, Hg produced the earliest and strongest response, with a lipid EC_10_ of 0.0706 mg L^−1^ (0.0651–0.0884) (Table 2). This value is only slightly higher than that of the corresponding fluorescence EC_10_ of 0.0581 mg L^−1^, indicating that metabolic reallocation toward neutral lipids begins at approximately the same concentration at which pigment-associated biomass starts to decline. This tight coupling between pigment impairment and lipid accumulation is consistent with combined effects of Hg on thiol-containing metabolic enzymes and pigment-associated proteins.

For As and Cr(VI), the relationships between lipid and fluorescence endpoints were more separated, revealing a sequence in which pigment-level disruption was followed by metabolic reprogramming. In *C. closterium*, the lipid EC_10_ values for As and Cr(VI) were 1.691 mg L^−1^ (1.560–4.069) and 2.623 mg L^−1^ (2.545–2.810), respectively (Table 2). These thresholds fell between the corresponding fluorescence EC_10_ and EC_50_ values for each metal (Table 1), indicating that pigment-associated fluorescence declined at lower concentrations, whereas measurable lipid accumulation required somewhat higher exposure levels. This separation is consistent with a two-stage response in which early photosynthetic stress is followed by carbon reallocation once oxidative burden exceeds compensatory capacity.

For Cd, lipid accumulation began at an EC_10_ of 2.544 mg L^−1^ (2.089–2.998), whereas the fluorescence EC_10_ and EC_50_ values were 2.826 mg L^−1^ and 5.416 mg L^−1^, respectively. This pattern suggests that pigment-associated responses and lipid-body formation occur over overlapping intermediate concentration ranges, with fluorescence inhibition preceding but not greatly outpacing the onset of metabolic reallocation.

In *T. weissflogii*, lipid-body accumulation generally required higher metal concentrations to reach the EC_10_ threshold than in *C. closterium*, although the specific patterns varied among metals (Table 2). For several metals, including Cr(III), Cu, and As, lipid responses were detectable at low concentrations, with EC_10_ values of 0.6630, 0.0311, and 0.028 mg L^−1^, respectively. In contrast, Cd and Ni required substantially higher exposures to elicit lipid accumulation, with EC_10_ values of 10.109 and 6.2547 mg L^−1^. For Cr(VI), Sb, Pb, Zn, and Hg, no resolvable lipid EC_10_ values could be obtained within the tested range, reflecting either minimal or highly variable lipid-body responses in the planktonic species. Overall, these results indicate that *C. closterium* initiates measurable metabolic reallocation at lower external concentrations than *T. weissflogii*, consistent with a more sensitive and conservative physiological stress response in the benthic diatom.

**Figure 4:**
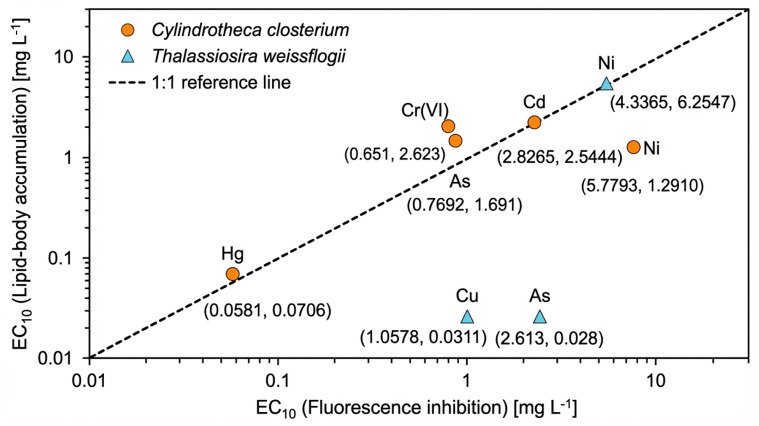
Comparison of EC_10_ values for fluorescence inhibition (*x*-axis) and lipid-body accumulation (*y*-axis) in two marine diatoms. Orange circles represent *Cylindrotheca closterium* and blue triangles represent *Thalassiosira weissflogii*. The dashed 1:1 line indicates equal sensitivity of the two endpoints.

When fluorescence and lipid endpoints were compared directly, metals differed markedly in whether metabolic or pigment-associated responses were more sensitive (Figure 4). Hg was the only metal for which lipid-body accumulation and fluorescence inhibition occurred at nearly identical concentrations, indicating close coupling between metabolic disturbance and pigment impairment. For Cr(VI) in *C. closterium*, lipid EC_10_ values also fell below the corresponding fluorescence EC_10_, suggesting that metabolic reorganization can begin prior to detectable pigment loss.

For most other metals, however, lipid EC_10_ values exceeded fluorescence EC_10_ values, indicating that pigment-associated fluorescence was the more sensitive endpoint under the present exposure conditions. This pattern was observed in *C. closterium* for As and Cd, both of which plotted above the 1:1 reference line in Figure 4. In these cases, lipid-body formation occurred only after appreciable inhibition of fluorescence, implying that metabolic reallocation represents a later stage of cellular stress.

In contrast, an opposite pattern was observed in *T. weissflogii* for As and Cu, where lipid EC_10_ values were substantially lower than fluorescence EC_10_ values. These points fall well below the 1:1 line, indicating that lipid-body accumulation responds at lower exposure levels and may serve as an early-warning indicator of stress.

Ni showed species-dependent behavior, with near coupling in *T. weissflogii* but a clear shift toward higher lipid EC_10_ values in *C. closterium*. This distinction between metabolic-stress-dominated and biomass-proxy-dominated responses demonstrates that the two endpoints capture different positions along the stress-response sequence, enabling mechanistic discrimination among metals.

### 3.3. Comparison with Other Diatoms and Microalgae

A comprehensive summary of the EC_10_ and EC_50_ values obtained in this study across all toxicants and endpoints is presented in Table 3. Comparison of these values with previously reported toxicity data across marine and freshwater microalgae reveals both areas of agreement and notable divergences that reflect species-specific physiological and ecological characteristics. Among marine diatoms, several studies have reported Cu, Zn, and Cd EC_50_ values associated with growth inhibition in *Skeletonema costatum*, with representative 96 h EC_50_ values of 1.11 mg L^−1^ for Cu, 2.13 mg L^−1^ for Zn, and 6.84 mg L^−1^ for Cd [25]. These values fall below the fluorescence-derived EC_50_ estimates obtained here for *C. closterium* (e.g., >20 mg L^−1^ for Cd and >10 mg L^−1^ for Zn), suggesting that growth-based endpoints may be more conservative indicators of metal toxicity than pigment-associated fluorescence for certain metals. This observation is consistent with the fact that growth integrates the cumulative effects of stressors on multiple cellular processes over extended exposure periods.

A broader comparison with other diatom species reinforces this pattern of variability. For example, the marine diatom *Ditylum brightwellii* exhibits relatively high sensitivity to Ni, with EC_50_ values near 0.30 mg L^−1^ [36], whereas the estuarine diatom Coscinodiscus centralis shows a much lower EC_50_ of 0.10 mg L^−1^ toward As_2_O_3_ [27]. Likewise, growth-based EC_50_ values for Cu in other marine diatoms tend to fall in the low µg L^−1^ range, including *Cerataulina pelagica* (0.0056–0.0135 mg L^−1^) and *Phaeodactylum tricornutum* (0.0018–0.0073 mg L^−1^) [30]. These values are several orders of magnitude lower than the fluorescence-based EC values reported for *T. weissflogii* and *C. closterium* in the present study, further illustrating methodological and species-level differences in endpoint sensitivity.

Microalgal taxa outside the diatom lineage show an equally wide range of sensitivities. The marine green alga *Tetraselmis chuii* exhibits moderate sensitivity to Pb, with a 72 h EC_50_ of 2.66 mg L^−1^ [39]. Dinoflagellates likewise show substantial variation: *Alexandrium pacificum* displays EC_50_ values of 0.35 mg L^−1^ for Cu, 1.45 mg L^−1^ for Zn, and 1.07 mg L^−1^ for Cr [28], while *Cochlodinium polykrikoides* exhibits EC_50_ values of 12.74 mg L^−1^ for Cu and 46.71 mg L^−1^ for Pb [38]. These differences highlight the influence of cellular architecture, metal uptake pathways, and metabolic organization on metal toxicity across microalgal groups.

Freshwater diatoms and green microalgae provide an additional point of comparison. The freshwater diatom *Navicula pelliculosa* shows relatively low EC_50_ values for several metals, including 0.32 mg L^−1^ for Cu, 0.50 mg L^−1^ for Cd, and 0.10 mg L^−1^ for Ni [29], reflecting higher free-ion activity in freshwater systems. Similarly, the standard OECD test species *Pseudokirchneriella subcapitata* exhibits EC_50_ values well below 1 mg L^−1^ for multiple metals, including approximately 0.15 mg L^−1^ for Cu and 0.50 mg L^−1^ for Ni [4]. These contrasts align with expectations from metal speciation theory, where the high ionic strength and ligand-rich nature of seawater substantially reduce free metal activity and thereby apparent toxicity.

For highly potent metals, the present findings correspond well with available microalgal data. The EC_10_ and EC_50_ values for Hg in *C. closterium* and *T. weissflogii* (both <0.2 mg L^−1^) fall within the same order of magnitude as the 96 h growth EC_50_ of 0.0286 mg L^−1^ reported for the marine diatom *Odontella mobiliensis* [33]. These concordant values emphasize the pronounced susceptibility of diatom metabolic and pigment systems to Hg, irrespective of species-specific differences in habitat or physiology. Comparable patterns are observed for Cr(VI), where both diatoms in this study show low-mg L^−1^ EC_10_ values consistent with previously reported sensitivity ranges for marine phytoplankton.

Collectively, these comparisons indicate that the two diatoms examined here—*C. closterium* and *T. weissflogii*—fall within the middle-to-high sensitivity range of the broader microalgal toxicological spectrum. *C. closterium*, in particular, emerges as a relatively sensitive benthic indicator for Hg, As, and Cr(VI), supporting its utility in setting conservative ecological protection thresholds for metal contamination in coastal ecosystems. Nevertheless, cross-system differences in ionic strength, speciation, and metal–ligand interactions underscore the need for context-specific interpretation when comparing toxicity values across marine and freshwater taxa.

## 4. Discussion

### 4.1. General Mechanisms of Metal Toxicity and Their Reflection in the Current Results

**Figure 5:**
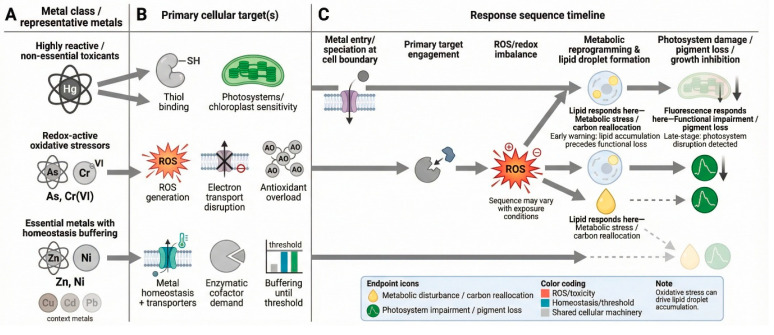
Mechanistic synthesis model linking metal targets to dual-endpoint response sequences. Conceptual model summarizing how representative metals engage primary cellular targets and trigger staged stress cascades in diatoms. Reactive toxicants such as Hg strongly interact with cellular thiols and photosynthetic machinery, leading to rapid redox imbalance and coupled changes in fluorescence and lipid-body accumulation. Redox-active stressors (As, Cr(VI)) induce oxidative stress and disrupt photosynthetic function while also promoting metabolic remodeling associated with lipid droplets, producing endpoint decoupling that enables metal-specific mechanistic discrimination. Essential metals (Zn, Ni) are buffered by homeostasis and typically exhibit high response thresholds. By mapping fluorescence inhibition to later-stage functional impairment and lipid accumulation to earlier metabolic disturbance, the dual-endpoint framework provides mechanistic interpretability beyond empirical toxicity ranking. Arrows indicate the direction and progression of cellular response sequences from metal exposure to downstream physiological and metabolic effects.

The mechanistic relationships linking metal class, primary cellular targets, and endpoint responses are synthesized in Figure 5. When considered alongside these mechanistic categories, the patterns observed in fluorescence inhibition and lipid-body accumulation across the two diatom species are coherent with well-established modes of metal action.

Hg represents a highly thiol-reactive toxicant capable of simultaneously targeting metabolic enzymes and photosynthetic proteins. Its strong affinity for cysteine residues leads to rapid inactivation of thiol-containing enzymes and disruption of photosystem components, while also promoting ROS formation and weakening antioxidant defenses. These combined effects manifest as near-simultaneous functional and metabolic impairment. This mechanistic expectation aligns closely with the experimental results: both *C. closterium* and *T. weissflogii* exhibited very low fluorescence EC_10_ values (0.0581 and 0.0760 mg L^−1^, respectively) and similarly low lipid EC_10_ values (~0.07 mg L^−1^). Such tight coupling between pigment loss and lipid-body induction supports the classification of Hg as a “fast-acting, strongly coupled” toxicant whose primary and downstream targets converge rapidly.

As and Cr(VI), in contrast, are classical redox-active stressors. Their toxicity arises from ROS overproduction, electron-transport disruption, and damage to nucleic acids and proteins. In *C. closterium*, fluorescence EC_10_ values for As and Cr(VI) (0.7692 and 0.651 mg L^−1^) were lower than their respective lipid EC_10_ values (1.691 and 2.623 mg L^−1^), indicating that photosystem structure and pigment synthesis are impaired before metabolic reallocation is triggered. This ordering is characteristic of a staged oxidative-stress response: diatom cells initially attempt to maintain photosystem integrity but activate neutral-lipid formation only when ROS accumulation overwhelms antioxidant capacity. Such endpoint separation provides mechanistic resolution by distinguishing early photophysiological disruption from later metabolic reprogramming.

Cd displays yet a different mechanistic profile. Although Cd can bind thiol groups, it does not participate directly in redox cycling. Instead, Cd interferes with membrane transporters, displaces essential metal cofactors, and induces the production of phytochelatins and metallothioneins. The fluorescence EC_10_ of *C. closterium* for Cd (2.8265 mg L^−1^) and its lipid EC_10_ (2.544 mg L^−1^) fall within an overlapping intermediate range, while fluorescence EC_50_ values remain comparatively high (>5 mg L^−1^). These patterns suggest that Cd elicits moderate pigment impacts and modest metabolic stress but only produces strong photophysiological impairment at concentrations high enough to saturate intracellular buffering systems. Thus, Cd triggers metabolic and pigment responses that are neither tightly coupled (as with Hg) nor strongly sequential (as with As and Cr(VI)), reflecting its role as a non-redox thiol-binding metal.

The patterns resolved by the two endpoints are consistent with the pathways outlined in Figure 5. Fluorescence inhibition tracked the onset of photosystem impairment, whereas lipid-body accumulation reflected earlier metabolic adjustments associated with oxidative or thiol-reactive stress. The relative positions of these thresholds differed among metals in ways that match their known modes of action, indicating that the endpoints capture distinct stages of the cellular response. In this framework, fluorescence and lipid accumulation provide complementary information on how diatoms reorganize carbon metabolism and photophysiology under metal exposure, offering a mechanistic basis for interpreting species- and metal-specific toxicity profiles.

### 4.2. Implications for Managing Diatom Communities and Setting Regulatory Thresholds

The observed differences in sensitivity between the two diatom species have direct implications for assessing metal impacts in coastal environments. *C. closterium* consistently showed lower EC_10_ values than *T. weissflogii* for several metals, including As, Cr(VI), Cd, and Pb, indicating that benthic diatoms may respond to metal exposure at earlier stages and therefore serve as conservative indicators of sediment-associated contamination. This species-level distinction aligns with evidence that benthic microalgae experience higher effective exposure due to boundary-layer processes and contact with metal-enriched porewater [41,42]. In operational terms, *C. closterium*-based EC_10_ thresholds could inform screening criteria for activities that mobilize sediment—such as dredging, land reclamation, or disposal of dredged material—where elevated porewater metal concentrations pose a risk to benthic primary producers.

A tiered monitoring framework could incorporate both endpoints used here. Fluorescence inhibition provides a rapid indication of functional impairment, whereas elevated lipid-body accumulation in the absence of fluorescence suppression would identify sublethal metabolic stress warranting further investigation. The use of metabolic biomarkers as early indicators of stress has been proposed for phytoplankton-based monitoring, particularly in systems experiencing episodic contaminant pulses [43]. Applying such a two-tiered strategy to benthic diatom communities would increase the sensitivity of routine assessments while avoiding unnecessary responses to transient or low-level disturbances.

From a regulatory perspective, the EC_10_ values obtained here fall within the milligram-per-liter range, well above the environmental quality standards (EQS) established under the EU Water Framework Directive for metals such as Cd, Ni, Pb, and Hg, which are typically set in the nanogram- to low microgram-per-liter range [44]. This disparity highlights that laboratory-derived thresholds should not be interpreted as replacements for cross-taxa regulatory criteria. Instead, they provide species- and habitat-specific information that can refine local management decisions, particularly in sediment-disturbance contexts where exposure conditions differ markedly from the water-column scenarios on which EQS values are based.

The relatively high sensitivity of *C. closterium* to Hg, As, and Cr(VI) suggests that benthic diatoms could contribute to the development of ecological “adjustment factors” that account for habitat-specific vulnerability in coastal risk assessment, an approach increasingly advocated in metal-specific guidance. Moreover, the sensitivity of the fluorescence endpoint to early pigment disruption, combined with the ability of lipid-body accumulation to detect upstream metabolic reallocation, indicates that these two endpoints can be incorporated into monitoring programs aimed at identifying emerging stress before community-level changes occur. For example, samples showing elevated lipid accumulation without concurrent fluorescence loss may signal metabolic strain in the absence of overt biomass reduction, prompting more detailed chemical or bioassay follow-up.

### 4.3. Future Directions and Research Needs

**Figure 6:**
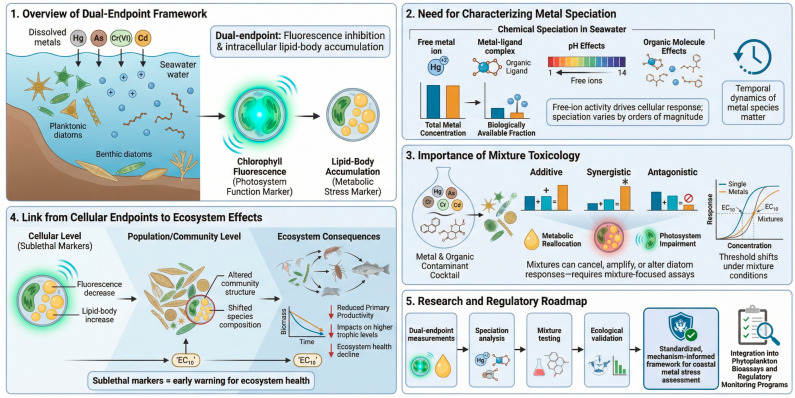
Conceptual roadmap for advancing dual-endpoint metal toxicity assessment in marine diatoms. Schematic overview summarizing key components of the dual-endpoint framework. (**1**) Fluorescence inhibition and intracellular lipid-body accumulation as complementary endpoints, (**2**) metal speciation processes influencing bioavailability, (**3**) mixture toxicity effects (additive, synergistic, antagonistic), (**4**) scaling of sublethal responses to population and ecosystem-level consequences, and (**5**) integration into a standardized, mechanism-informed framework for coastal metal stress assessment and regulatory monitoring. Arrows indicate the direction and progression of processes linking exposure, cellular responses, and ecological outcomes.

Although the dual-endpoint framework developed here provides a quantitative basis for interpreting metal-induced stress in benthic and planktonic diatoms, several areas require further investigation. Figure 6 summarizes these research and regulatory priorities within a broader conceptual roadmap.

A clear priority is the characterization of metal speciation and free-ion activity under exposure conditions. Because the biologically available fraction of a metal often departs substantially from its nominal concentration, resolving the temporal dynamics of species such as Hg, As, Cr(VI), and Cd in seawater would improve mechanistic interpretation of cases where lipid-body accumulation precedes—or lags behind—fluorescence inhibition. Recent studies highlight that free-ion activity can differ by orders of magnitude depending on ligand availability, pH, and organic complexation [45,46], underscoring the importance of integrating chemical speciation with cellular responses.

A second research direction concerns the role of multi-metal and metal–organic mixtures. Coastal systems frequently contain mixtures rather than isolated contaminants, and interactions among metals can be additive, synergistic, or antagonistic. Expanding dual-endpoint assays to controlled mixtures would clarify whether metabolic reallocation and photosystem impairment interact predictably across stressors. Advances in mixture toxicology for microalgae [47] show that such interactions can substantially alter threshold concentrations and modify response trajectories, emphasizing the need for mixture-focused experimental designs.

A third area involves linking sublethal cellular responses to ecological consequences. While fluorescence and lipid EC_10_ values capture early impairment under laboratory conditions, their implications for diatom community structure, primary productivity, and trophic interactions remain to be established. Field studies demonstrate that shifts in diatom physiology can precede measurable changes in biomass or species composition [48,49], suggesting that sublethal markers may offer predictive insight into ecosystem-level responses. Quantifying how metabolic and photophysiological stress propagate to community and food-web dynamics will help translate cellular endpoints into ecologically meaningful protection criteria.

These directions outline a pathway for refining the interpretive and operational value of dual-endpoint diatom assays. By coupling fluorescence and lipid-body measurements with speciation analysis, mixture testing, and ecological validation, it should be possible to develop a standardized, mechanism-informed framework for assessing metal stress in coastal systems. Such an approach would align with ongoing efforts to modernize phytoplankton bioassays and incorporate functional biomarkers into regulatory and monitoring programs.

## 5. Conclusions

This study quantified the toxic effects of ten environmentally relevant metals on two ecologically contrasting marine diatoms—benthic *Cylindrotheca closterium* and planktonic *Thalassiosira weissflogii*—using a dual-endpoint framework that couples chlorophyll fluorescence with Nile Red-based lipid-body measurements. Fluorescence revealed clear concentration–response patterns and identified Hg, As, and Cr(VI) as the most potent inhibitors of photosystem function. Across these metals, *C. closterium* consistently responded at lower concentrations than *T. weissflogii*, underscoring the heightened vulnerability of benthic diatoms to dissolved and particle-associated contaminants. Lipid-body accumulation frequently preceded detectable fluorescence inhibition—particularly for Hg, As, and Cd—indicating that metabolic reallocation and oxidative stress can serve as early markers of sublethal disturbance.

Comparison with published EC values for other diatoms and freshwater algae showed that the sensitivities observed here fall within the upper range of microalgal responses, with *C. closterium* aligning with the most sensitive taxa reported for redox-active and thiol-reactive metals. These findings emphasize that benthic diatoms should be explicitly represented in coastal risk assessments, rather than relying solely on data from planktonic or freshwater species. The strong correspondence between fluorescence-based EC values and growth-derived toxicity benchmarks for metals such as Cu further demonstrates that fluorescence can serve as an operationally efficient endpoint compatible with standardized, automation-ready toxicity assays.

By pairing a rapid biomass proxy with a mechanistically informative metabolic marker, the dual-endpoint approach applied here resolves species-specific sensitivity differences, metal-specific toxicity fingerprints, and the sequence from metabolic stress to functional impairment. The resulting EC_10_ matrix across ten metals and two diatom guilds provides a quantitatively robust foundation for species sensitivity distribution (SSD) modeling, derivation of site-specific trigger values, and the development of tiered decision frameworks that integrate screening and assessment phases. Ultimately, these findings support the incorporation of diatom-based bioassays into regulatory monitoring and highlight the functional relevance of diatoms as early sentinels of metal stress in coastal ecosystems.

## Figures and Tables

**Table 1:** EC_10_ and EC_50_ values (mg L^−1^) for fluorescence-based biomass inhibition and lipid-body accumulation in two marine diatoms after 48 h exposure. Values were derived from three-parameter logistic models with 95% confidence intervals (CI). Lower EC values indicate higher sensitivity.

Metals	*C. closterium*		*T. weissflogii*	
	EC_10_	EC_50_	EC_10_	EC_50_
As	0.7692 (0.5273–1.0807)	2.5585 (2.3755–2.6835)	2.613 (0.759–8.494)	8.291 (6.916–26.335)
Cd	2.8265 (2.337–3.366)	5.4160 (5.258–5.593)	>20	>20
Cr(III)	>10	>10	–	–
Cr(VI)	0.651 (0.426–1.266)	4.588 (4.201–6.041)	1.5596 (0.0280–4.3964)	>20
Cu	>2	>2	1.0578 (1.2225–0.9015)	1.8877 *
Hg	0.0581 (0.0384–0.0692)	0.1554 (0.1414–0.1731)	0.0760 (0.0403–0.0790)	0.1235 *
Ni	5.7793 (5324–7.2618)	>10	4.3365 (3.4124–5.3663)	>10
Pb	>10	>10	1.2061 (0.9155–7.7380)	>10
Sb	1.394 (1.190–1.736)	6.660 (4.408–10.075)	>50	>50
Zn	1.4192 (1.3640–1.4640)	>10	>10	>10

“>” indicates that the EC value exceeded the highest test concentration within the 48 h exposure range. * EC values without confidence intervals could not be reliably estimated due to lack of measurable inhibition or insufficient model fit.

**Table 2:** EC_10_ and EC_50_ values (mg L^−1^) for lipid-body accumulation in *Cylindrotheca closterium* and *Thalassiosira weissflogii* after 48 h metal exposure. Values were derived from three-parameter logistic models with 95% confidence intervals (CIs). Lower EC values indicate higher sensitivity.

Metals	*C. closterium*		*T. weissflogii*	
	EC_10_	EC_50_	EC_10_	EC_50_
As	1.691 (1.560–4.069)	5.469 (4.387–6.267)	0.028 (0.020–0.044)	11.697 (6.924–16.027)
Cd	2.5444 (2.0892–2.9989)	>20	10.109 (10.150–10.752)	15.445 (12.867–17.564)
Cr(III)	ND	ND	0.6630 (0.6273–0.7366)	7.2326 *
Cr(VI)	2.623 (2.545–2.810)	15.792 (11.883–19.451)	>20	>20
Cu	>2	>2	0.0311	>2
Hg	0.0706 (0.0651–0.0884)	>1	ND	ND
Ni	1.2910 *	>10	6.2547 *	>10
Pb	>10	>10	ND	ND
Sb	>50	>50	>50	>50
Zn	>10	>10	ND	ND

“>” indicates that the EC value exceeded the highest test concentration within the 48 h exposure range. * EC values without confidence intervals could not be reliably estimated due to lack of measurable inhibition or insufficient model fit.

**Table 3:** Reported EC_50_ values (mg L^−1^) for metal toxicity in marine and freshwater microalgae. Values were compiled from published studies. Exposure duration, endpoint, and reference are shown to highlight methodological variability among studies. ND indicates that EC_50_ values were not reported or could not be calculated.

Metals	Species	EC_50_ (mg L^−1^)	95% CI	Exposure Time	Endpoint	Reference
As(V)	*Skeletonema costatum*	0.0028	±0.0009	72 h	Population growth	[26]
As_2_O_3_	*Coscinodiscus centralis*	0.1	–	4 d	Population growth	[27]
Cd	*Palatinus apiculatus*	1.35	–	72 h	Cell growth	[28]
Cd	*Navicula pelliculosa*	0.5	0.47–0.54	4 d	Free ion activity	[29]
Cd	*Alexandrium pacificum*	4.75	±0.8	72 h	Cell density	[28]
Cr	*Alexandrium pacificum*	1.07	±0.04	72 h	Cell density	[28]
Cu	*Navicula pelliculosa*	0.32	0.30–0.33	4 d	Free ion activity	[29]
Cu	*Cerataulina pelagica*	0.0135	–	3 d	Cell growth rate	[30]
Cu	*Cerataulina pelagica*	0.0056	–	3 d	Cell growth rate	[30]
Cu	*Phaeodactylum tricornutum*	0.0018	–	3 d	Cell growth rate	[30]
Cu	*Phaeodactylum tricornutum*	0.0073	–	3 d	Cell growth rate	[30]
Cu	*Levanderina fissa*	0.0046	–	3 d	Cell growth rate	[30]
Cu	*Prorocentrum nanum*	0.0317	–	3 d	Cell growth rate	[30]
Cu	*Prorocentrum nanum*	0.0437	–	3 d	Cell growth rate	[30]
Cu	*Alexandrium pacificum*	0.35	±0.02	72 h	Cell density	[28]
Cu	*Chaetoceros tenuissimus*	9.87	±1.0	48 h	Cell density	[31]
Cu	*Chaetoceros tenuissimus*	7.66	±0.9	48 h	Cell density	[31]
Cu	*Chaetoceros tenuissimus*	11.45	±0.5	48 h	Cell density	[31]
Cu	*Cochlodinium polykrikoides*	12.74	4.515–4.819	72 h	Cell density	[32]
Hg	*Odontella mobiliensis*	0.0286		96 h	Growth inhibition	[33]
HgCl_2_	*Pseudokirchneriella subcapitata*	0.039–0.06		72 h	Growth inhibition	[34]
HgCl_2_	*Chlorella vulgaris*	0.002		72 h	Growth inhibition	[35]
Ni	*Navicula pelliculosa*	0.1	0.091–0.11	4 d	Free ion activity	[29]
Ni	*Alexandrium pacificum*	0.85	±0.10	72 h	Cell density	[28]
Ni	*Ditylum brightwellii*	0.3	–	5 d	Population growth	[36]
NiCl_2_	*Ditylum brightwellii*	0.3	–	5 d	Population growth	[37]
Pb	*Cochlodinium polykrikoides*	46.71	3.986–4.566	72 h	Cell density	[38]
Pb	*Tetraselmis chuii*	2.66	–	72 h	Cell density	[39]
Pb	*Navicula incerta*	10.96	–	96 h	Cell density	[39]
SbCl_3_	*Scenedesmus subspicatus*	10.685				[40]
SbCl_3_	*Chlorococcum infusionum*	7.8				[40]
Zn	*Navicula pelliculosa*	3.4	3.1–3.8	4 d	Free ion activity	[29]
Zn	*Alexandrium pacificum*	1.45	±0.22	72 h	Cell density	[28]

## Data Availability

The datasets generated and analyzed during the current study are not publicly archived but are available from the corresponding author upon reasonable request.

## Abbreviations

The following abbreviations are used in this manuscript:

EC_10_	Effective Concentration 10
EC_50_	Effective Concentration 50
EQS	Environmental Quality Standard
ISO	International Organization for Standardization
OECD	Organization for Economic Co-operation and Development
ROS	Reactive Oxygen Species
NR	Nile Red
FU	Fluorescence Units
SSD	Species Sensitivity Distribution
LOEC	Lowest Observed Effect Concentration
NOEC	No Observed Effect Concentration
DMSO	Dimethyl sulfoxide
PTFE	Polytetrafluoroethylene
PFD	Photon Flux Density
